# Prevention, Diagnosis and Management of Post-Surgical Mediastinitis in Adults Consensus Guidelines of the Spanish Society of Cardiovascular Infections (*SEICAV*), the Spanish Society of Thoracic and Cardiovascular Surgery (*SECTCV*) and the Biomedical Research Centre Network for Respiratory Diseases (*CIBERES*)

**DOI:** 10.3390/jcm10235566

**Published:** 2021-11-26

**Authors:** Emilio Bouza, Arístides de Alarcón, María Carmen Fariñas, Juan Gálvez, Miguel Ángel Goenaga, Francisco Gutiérrez-Díez, Javier Hortal, José Lasso, Carlos A. Mestres, José M. Miró, Enrique Navas, Mercedes Nieto, Antonio Parra, Enrique Pérez de la Sota, Hugo Rodríguez-Abella, Marta Rodríguez-Créixems, Jorge Rodríguez-Roda, Gemma Sánchez Espín, Dolores Sousa, Carlos Velasco García de Sierra, Patricia Muñoz, Martha Kestler

**Affiliations:** 1 Clinical Microbiology and Infectious Diseases Department, Gregorio Marañon University Hospital, Gregorio Marañon Health Research Institute, Complutense University of Madrid, CIBER of Respiratory Diseases—CIBERES, 28007 Madrid, Spain; emilio.bouza@salud.madrid.org (E.B.); mrcreixems@salud.madrid.org (M.R.-C.); patricia.munoz.garcia@salud.madrid.org (P.M.); 2 Virgen del Rocío University Hospital, 41013 Seville, Spain; caristo.alarcon.sspa@juntadeandalucia.es; 3 Marqués de Valdecilla University Hospital, 39008 Santander, Spain; mcfarinas@humv.es; 4 Virgen Macarena University Hospital, 41009 Seville, Spain; jga@us.es; 5 Donosti Hospital, 20014 Donostia-San Sebastian, Spain; miguelangel.goenagasanchez@osakidetza.eus; 6 Cardiovascular Surgery Department, Marques de Valdecilla University Hospital, 39008 Santander, Cantabria, Spain; josefrancisco.gutierrez@scsalud.es; 7 Anesthesia and Intensive Care Department, Gregorio Marañon University Hospital, 28007 Madrid, Spain; franciscojavier.hortal@salud.madrid.org; 8 Plastic Surgery Department, Gregorio Marañon University Hospital, 28007 Madrid, Spain; josemaria.lasso@salud.madrid.org; 9 Department of Cardiac Surgery, University Hospital Zurich, 8091 Zurich, Switzerland; carlos.mestres@usz.ch; 10 Infectious Diseases Services, Hospital Clinic-IDIBAPS, University of Barcelona, 08036 Barcelona, Spain; josemaria@miromoreno.org; 11 Infectious Diseases Department, Ramón y Cajal University Hospital, 28034 Madrid, Spain; enavase@salud.madrid.org; 12 Cardiovascular Unit, Intensive Care Department, San Carlos Clinical Hospital, 28040 Madrid, Spain; mernietoc@salud.madrid.org; 13 Department of Radiology, Marquez de Valdecilla University Hospital, 39008 Santander, Cantabria, Spain; joseantonio.parra@scsalud.es; 14 Cardiac Surgery Department, Hospital 12 de Octubre, 28041 Madrid, Spain; epsccv@arrakis.es; 15 Cardiac Surgery Department, Gregorio Marañon University Hospital, 28007 Madrid, Spain; rhugorodriguez@salud.madrid.org; 16 Cardiac Surgery Department, Ramón y Cajal Hospital, 28034 Madrid, Spain; jorge.rodriguez-roda@salud.madrid.org; 17 Heart Clinical Management Unit, Virgen de la Victoria University Hospital, 29006 Malaga, Spain; gemma.sanchez.sspa@juntadeandalucia.es; 18 Infectious Diseases Department, A Coruña Hospital Complex, 15006 A Coruña, Spain; dsoureg@sergas.es; 19 Cardiac Surgery Department, A Coruña Hospital Complex, 15006 A Coruña, Spain; carlos.velasco.garcia.de.sierra@sergas.es

**Keywords:** mediastinitis, sternal wound infections, post-surgical mediastinitis, cardiac surgery, infection, surgical wound infection

## Abstract

This is a consensus document of the Spanish Society of Cardiovascular Infections (*SEICAV*), the Spanish Society of Thoracic and Cardiovascular Surgery (*SECTCV*) and the Biomedical Research Centre Network for Respiratory Diseases (*CIBERES*). These three entities have brought together a multidisciplinary group of experts that includes anaesthesiologists, cardiac and cardiothoracic surgeons, clinical microbiologists, infectious diseases and intensive care specialists, internal medicine doctors and radiologists. Despite the clinical and economic consequences of sternal wound infections, to date, there are no specific guidelines for the prevention, diagnosis and management of mediastinitis based on a multidisciplinary consensus. The purpose of the present document is to provide evidence-based guidance on the most effective diagnosis and management of patients who have experienced or are at risk of developing a post-surgical mediastinitis infection in order to optimise patient outcomes and the process of care. The intended users of the document are health care providers who help patients make decisions regarding their treatment, aiming to optimise the benefits and minimise any harm as well as the workload.

## 1. Introduction

Post-surgical mediastinitis (PSM) after cardiac surgery (CS) is defined as a deep sternal wound infection (SWI) with sternal osteomyelitis with or without infected retrosternal space and is associated high morbidity and mortality [1]. The incidence of PSM varies from 1 to 5%, and rates >2% are generally indicators of poor quality of care in cardiovascular surgery [2,3]. Cardiac surgery patients are frequently frail elderly subjects with many comorbidities and are, thus, predisposed to postoperative complications [4]. Many aspects regarding prevention, diagnosis and management of PSM are currently under discussion between different work groups, each with its own approach.

There is little information on the best prevention, diagnosis and management of PSM and it is scattered in the literature. Furthermore, current clinical practices are not always well supported by the medical literature. A critical review of the available information is essential, aiming to provide the best guidance to those interested and committed to this pathology, particularly when, up to now, there is no consensus document by any Spanish scientific society.

## 2. Scope and Purpose

This is a consensus document of the Spanish Society of Cardiovascular Infections (*SEICAV*), the Spanish Society of Thoracic and Cardiovascular Surgery (*SECTCV*) and the Biomedical Research Centre Network for Respiratory Diseases (*CIBERES*). These three entities have brought together a multidisciplinary group of experts that includes anaesthesiologists, cardiac and cardiothoracic surgeons, clinical microbiology, infectious diseases and intensive care specialists, internal medicine doctors and radiologists. Despite the clinical and economic consequences of sternal wound infections, to date, there are no specific guidelines for the prevention, diagnosis and management of PSM based on a multidisciplinary consensus. The purpose of the present document is to provide evidence-based guidance on the most effective diagnosis and management of patients who have experienced or are at risk of developing a mediastinitis in order to optimise patient outcomes and the process of care.

The intended users of the document are health care providers who help patients make decisions regarding their treatment, aiming to optimise the benefits and minimise any harm as well as the workload.

## 3. Materials and Methods

The work group formulated a set of questions, mainly in the Population, Intervention, Comparison and Outcome (PICO) framework. Each question was assigned to a pair of independent reviewers, who were asked to conduct a systematic search of the medical literature, using the following search strategy: the Cochrane Plus Library (UK), Medline/PUBMED (National Library of Medicine, USA), EMBASE (Elsevier, The Netherlands), Scopus (Elsevier) and the Trip database (UK) in the period between 1970 and June 2021. The results of the searches were thoroughly reviewed by panellists, after which a selection and evaluation of relevant articles was carried out. Evidence summaries for each question were prepared by the panel members using the Grading of Recommendations Assessment, Development and Evaluation (GRADE) format was used to write the recommendations and/or to grade the strength of the recommendations [5]. Evidence summaries were discussed and reviewed by all committee members and edited, as appropriate. Once the analyses were completed, the panellists presented their data and findings to the whole panel for deliberation and drafting of recommendations.

Reviewers were asked to rate the findings in the literature based on the level of evidence extracted from the articles and classify the recommendations by grade of evidence. The National Institute for Health and Care Excellence (NICE) method—an adaptation of the Scottish Intercollegiate Guidelines Network (SIGN) [6] for intervention studies—was agreed upon. This scale proposes two attributes to assess the quality of the available scientific evidence (levels of evidence): study design and risk of bias. A rating between 1 and 4 was used to rank the design of the studies.

To assess the risk of bias, ++, + and − were used to indicate to which extent the key criteria were linked to the potential risk (Table 1).

There is an ongoing need for research on almost every topic considered in this guideline. However, *research needs* were contemplated for recommendations when the panellists considered that the need was particularly acute. There is a lack of high quality of evidence for many of the recommendations. Strong recommendations have sometimes been made in the setting of low quality of evidence, when it was believed that most individuals would want the recommended course of action and that most well-informed physicians would agree, despite the low quality of the evidence.

Recommendations were classified as either “strong” or “weak” (conditional) considering the GRADE approach [5] (Table 2). The terms *recommend* and *suggest* indicate strong or weak recommendations, respectively.

The draft of the document was prepared by the coordinator of the scientific committee using the information received from the work groups and suggestions of the *SEICAV* 2019 assembly meeting participants. Prior to its final approval, the document was made available to all members of the Scientific Committee for further information and comments. All panel members took part in the preparation of the guideline and approved the final recommendations. The definitive version was reviewed and submitted to the *SEICAV* domain for further input and application.

The document was structured in four different sections: Prevention, Diagnosis, Surgical Management and Medical Management. A summary table has been added at the end of the document.

## 4. Prevention

### 4.1. Does Preoperative Control of Hyperglycaemia in Adult Patients Reduce the Risk of Mediastinitis?

Optimising preoperative glycaemic control is recommended in diabetic patients with high preoperative HbA1c levels (>6.5–7%) to reduce the risk of mediastinitis.

Evidence level 2++. Strong recommendation, moderate quality of evidence.

Perioperative hyperglycaemia has been shown to be associated with an increased risk of major adverse events following cardiac surgery, particularly in cases of deep surgical wound infection (SWI) [7,8]. Postoperative glycaemic variability increases in patients with poor preoperative glycaemic control. The American Diabetes Association recommends the use of A1c blood glycosylated haemoglobin (HbA1c) as a method to assess long-term glycaemic control in diabetic patients [9]. Preoperative HbA1C measures control of blood glucose levels over the preceding 3 to 4 months. Efforts to optimise glucose control prior to surgery, especially in patients with preoperative HbA1c > 6.5–7%, provide a strategy to reduce postoperative SWI after cardiac surgery [10,11].

In patients with hyperglycaemia requiring urgent surgery, intravenous perioperative insulin infusion is the most effective method of rapidly achieving glycaemic control. The Society of Thoracic Surgeons (STS) currently recommends maintaining perioperative glucose levels < 180 mg/dL in patients undergoing cardiac surgery [12].

### 4.2. Does Smoking Cessation before Surgery Reduce the Risk of Postoperative Mediastinitis?

Patients should be encouraged to stop smoking at least 30 days before cardiac surgery.

Evidence level 2+. Strong recommendation, moderate quality of evidence.

Smoking cessation has consistently been shown to provide important benefits in reducing complications in patients undergoing surgery [13]. Results of different studies support the recommendation to stop smoking for at least one month before cardiac surgery to improve postoperative outcomes and, in particular, to reduce the risk of postoperative pulmonary complications [14,15].

In a large prospective study, Nagachinta et al. found that smoking was an independent risk factor for mediastinal infection after cardiac surgery [16]. In a single-centre retrospective cohort study, Jones et al. report significant reductions in terms of pulmonary complications (6.8 vs. 11%, *p* = 0.01), readmission into the intensive care unit (ICU) (4.0 vs. 6.9%, *p* = 0.03) and infection (22.0 vs. 31.8%, *p* < 0.001), in non-smokers [17].

Quitting smoking for at least 30 days before surgery reduces the risk of PSM.

### 4.3. Does Weight Loss Reduce the Risk of Postoperative Mediastinitis in Obese or Overweight Adult Patients?

We recommend that obese or overweight patients should be encouraged to lose weight before surgery; we also recommend adjusting prophylactic antimicrobials doses, reinforcing the preparation of the surgical field and ensuring a very stable wound closure to avoid dehiscence, besides systematic closure with a NPWT device.

Evidence level 2++. Strong recommendation, moderate quality of evidence.

Obesity, defined as a body mass index (BMI) > 30, is a well-recognised independent risk factor for PSM, as has been demonstrated in at least 20 clinical studies [18,19,20,21,22,23,24,25,26,27,28,29,30,31,32,33,34,35,36,37,38,39,40,41,42,43,44,45]. Furthermore, obesity is one of the variables included in the scores used to stratify the risk of PSM [20,28,29,43,44,46,47,48,49]. The increased risk is proportional to excess BMI. Therefore, as it is one of the few potentially modifiable risk factors, overweight control is recommended before surgery whenever possible. However, there are insufficient data available to support the decision to delay a necessary surgery until sufficient weight loss is achieved. If cardiac surgery is performed on an obese patient, it is essential to adjust the dose of prophylactic antimicrobials, perform thorough preparation of the surgical field, and reinforce wound closure to prevent dehiscence. Some authors recommend systematic closure with negative pressure wound therapy (NPWT) [33,50].

### 4.4. Which Non-Antibiotic Measures Should Be Recommended to Prevent Postoperative Mediastinitis?

Existing evidence does not support the benefit of preoperative chlorhexidine showers over other products.

Evidence level 1+. Strong recommendation, high quality of evidence.

When hair removal is considered necessary, we recommend the use of a depilatory cream or an electric razor, never a blade.

Evidence level 1+. Strong recommendation, high quality of evidence.

In patients who will undergo cardiac surgery, a wide range of prophylactic regimens based on non-antibiotic measures are currently used. Among the most frequent ones are those aimed at preparing the surgical area either using disinfectant solutions and/or depilation. In one series, preoperative chlorhexidine showers or baths reduced bacterial colonisation of the skin, but were not associated with a clear reduction in SWI [51]. A systematic review of 20 randomised and non-randomised studies with 9520 patients included only 1 study in cardiac surgery patients that was inconclusive [52]. In a Cochrane review with over 10,500 patients, chlorhexidine was not clearly superior to placebo or regular soap [53]. New strategies include skin preparation with a 2% chlorhexidine gluconate cloth, which reduces surgical wound infection rates in patients undergoing orthopaedic surgery [54,55].

Shaving with cutting materials causes mild erosions to the patient that result in the accumulation of blood, facilitating bacterial overgrowth at human body temperature [56,57]. When depilation is required, the use of clipping is preferred. In a 2011 Cochrane review that included 14 studies, no significant differences in IHQ were observed between shaved and unshaved patients in 6 of the publications [58]. In three studies with 1340 subjects, shaved patients had more infections than those who underwent a haircut. Comparisons between shaving and the use of depilatory creams revealed no significant differences, although the studies were underpowered.

### 4.5. Should Staphylococcus aureus Nasal Carriage Be Assessed in Patients Undergoing Cardiac Surgery? Is it Effective to Eradicate This Pathogen in Positive Cases?

We recommend knowing the state of *S. aureus* nasal carriage and proceed with its eradication if possible or time allowable in positive patients before cardiac surgery.

Evidence level 1−. Strong recommendation, moderate quality of evidence.

*S. aureus* is a major nosocomial pathogen worldwide [59]. *S. aureus* infections may have serious consequences—including SWIs—which consequently delay healing, extend hospital stay, increase antibiotic use, cause unnecessary pain, increase hospital costs, and may lead to the need for further intervention or even cause death.

Since the consequences of these infections may be extremely serious, effective prevention strategies are necessary. More than 80% of *S. aureus* infections are caused by the patients’ own colonising bacteria [60,61]. *S. aureus* colonises the skin and mucous membranes of humans, the nose being the most common site [62]. Currently, the presence of *S. aureus* in the nose is considered a well-defined risk factor for subsequent infection. Thus, it is recommended to know the nasal carrier status in all patients who will undergo MHS. The most common risk factor for increased likelihood of post-surgical staphylococcal infections, including mediastinitis, is sensitive or/and methicillin-resistant *S. aureus* nasal carriage [63,64,65,66]. In some studies, decolonisation before clean surgery has been associated with a reduction in long-term mortality [67]. A prospective, randomised, double-blind, placebo-controlled clinical trial conducted in Amsterdam, which included 991 patients undergoing elective cardiothoracic surgery and compared oropharyngeal rinse and nasal ointment containing either chlorhexidine gluconate or placebo, showed that the incidence of nosocomial infection in the chlorhexidine gluconate group and placebo group was 19.8% and 26.2%, respectively (absolute risk reduction (ARR), 6.4%; 95% confidence interval (CI), 1.1–11.7%; *p* = 0.002). In particular, lower respiratory tract infections and deep surgical site infections were less common in the chlorhexidine gluconate group than in the placebo group (ARR, 6.5%; 95% CI, 2.3–10.7%; *p* = 0.002; and 3.2%; 95% CI, 0.9–5.5%; *p* = 0.002, respectively).

Although some mupirocin and chlorhexidine resistance has been reported, results in a large sample of MRSA isolates collected during the REDUCE-MRSA trial reported that decreased susceptibility to Chlorhexidine (CHG), as measured by CHG MICs and carriage of qacA or qacB, was rare and was similar in frequency among MRSA isolates identified in decolonisation arms and in the screening and isolation arm. On the other hand, the prevalence of mupirocin resistance at baseline was moderate (7.1% LLMR and 7.5% HLMR), and the odds of mupirocin resistance during the intervention versus the baseline period did not differ between the targeted or universal decolonisation arms. To date, mupirocin and chlorhexidine resistance should not be of concern, but periodic surveillance studies are recommended.

### 4.6. What Is the Best Time and Technique to Assess S. aureus Carriage in Adult Patients Who Will Undergo Cardiac Surgery?

We recommend the evaluation of *S. aureus* nasal carriage within 15 days prior to cardiac surgery.

Evidence level 1++. Strong recommendation, moderate quality of evidence.

PCR-based techniques are recommended when a rapid screening method is required due to its high negative predictive value.

Evidence level 1++. Strong recommendation, moderate quality of evidence.

Assessing *S. aureus* carriage should ideally be performed within two weeks prior to surgery, since recolonisation is common in patients who had previously been treated for the same diagnosis [68]. However, it is often complicated to coordinate the time between the pre-surgical evaluation and the surgery. Recent studies report that nasal cultures of up to 30% [69] of the patients undergoing MHS are positive for *S. aureus* after leaving the operating room. Moreover, 37% of cardiac surgeries are urgent [70] with no time for culture and decolonisation.

The most common technique to determine carriage in nose cultures is by swabbing both nostrils and placing the samples in transport media. Cultures from other sites (groin, armpit, rectum, etc.) are not recommended [71]. Following swabbing, samples are cultivated either in blood agar or chromogenic medium to facilitate the detection of methicillin-resistant *S. aureus* (MRSA) and are considered negative after an incubation time of 72 h [71]. In cases with colonies suspected of being *S. aureus*, a definitive identification/antimicrobial susceptibility test must be carried out. This process may last between 3 and 6 days.

Molecular polymerase chain reaction (PCR) techniques allow much faster detection (1.5 h) and provide information on whether the microorganism is MSSA or MRSA. Simple techniques are commercially available, although the cost is much higher than that of a regular culture [72]. The main problem with this is that the isolated meaning of the presence of DNA is unknown in patients with negative culture and that if both techniques are not performed, there will be no isolates for antimicrobial susceptibility or molecular epidemiology analyses.

To date, there is no evidence that supports the usefulness of post-decolonisation verification sampling.

### 4.7. Which Is the Drug of Choice for Nasal Decontamination of S. aureus Carriers? Is Universal Prophylaxis Preferable?

We recommend topical mupirocin for nasal decontamination in combination with chlorhexidine for skin decontamination.

Evidence level 1+. Strong recommendation, high quality of evidence.

We suggest systematic decontamination in patients in whom nasal carrier status cannot be assessed in a timely manner.

Evidence level 3. Strong recommendation, low quality of evidence 

Antibiotics or topical antiseptics are recognised methods for *S. aureus* decolonisation. Mupirocin ointment is one of the most popular antibiotics in clinical practice. It is often used to eradicate *S. aureus* because of its microbiological efficacy, safety and low cost [59]. Other products, such as neomycin and Octenisan, have also been used; however, specific evidence for cardiac surgery patients is lacking.

Compared to antibiotics, antiseptics often target a wide range of microorganisms and may reduce the presence of other co-pathogens [73]. Antiseptics usually work without damaging the tissue, so they can be used on intact skin and certain types of open wounds [74]. The antiseptic chlorhexidine is effective against a wide range of Gram-positive and Gram-negative bacteria, lipophilic viruses and yeasts [75]. Depending on its concentration, it may have bactericidal or bacteriostatic activity. In topical applications, it has been shown to have the unique ability to bind to proteins in human tissues, e.g., skin and mucous membranes, with limited absorption throughout the body. Protein-bound chlorhexidine is slowly released, leading to prolonged activity [76].

Doebbeling et al. found that mupirocin is effective in rapidly removing *S. aureus* from the nose, although early nasal recolonisation is common [77]. A few well-powered randomised clinical trials regarding this topic have achieved statistical significance [66,78,79]. Overall, these trials suggest that nasal *S. aureus* decolonisation is beneficial in patients undergoing major surgery or prolonged stays in ICUs [65,80].

In patients from whom it is not possible to have information on the nasal carrier status of *S. aureus* before cardiac surgery, decolonisation is recommended until the result is known.

### 4.8. In Adult Patients Undergoing Cardiac Surgery through Median Sternotomy, Does Skin Preparation with Chlorhexidine Reduce the Risk of Post-Surgical Mediastinitis in Comparison to Povidone-Based Preparations?

We recommend chlorhexidine over povidone-based preparations for skin preparation in cardiac surgery.

Evidence level 2+. Strong recommendation, moderate quality of evidence.

The action of chlorhexidine (CH) or povidone iodine (PI) is slower and more superficial than that of alcohol. Moreover, CH persists in the skin for a significantly longer period. Hence, the use of alcoholic chlorhexidine (at CH concentrations greater than 0.5%) has been invoked because it combines the fast microbicide action of alcohol and the residual activity (persistence) of CH in the skin. The different formulations and application strategies of both compounds make it difficult to draw conclusions from studies addressing this question. A Cochrane Systematic Review carried out in 2015 [81] compared several antiseptics in 13 studies, from which 5 assessed the action of CH and PI, although not in cardiac surgery. Compounds that included alcohol (mainly 4% CH in 70% alcohol) seemed to be the most effective, although the evidence was low.

Results from randomised trials favour chlorhexidine. However, these trials did not focus on MHS [82,83,84,85]. They include different types of clean and clean-contaminated interventions, e.g., in clean non-abdominal surgeries, less infection with the use of CH was found.

In two of the trials, one of the above-mentioned antiseptics was included and compared against another compound. In a German record of nearly 3000 patients, a comparison between CH combined with isopropyl alcohol (IPA) against IPA alone was carried out and, although the combination resulted in a lower number of mediastinitis cases than with IPA alone, non-randomisation and different alcohol concentrations between the two preparations do not provide much strength to the study [84].

A randomised three-arm study in an American centre [86] compared four different skin preparation strategies (pre-wash and/or paint) with aqueous iodine povidone and other alcoholic iodophors, and found there was no difference between the preparation strategies, although there was a trend towards fewer infections with aqueous preparations.

In a randomised study, Stevens et al. assessed the possible benefit of adding a plastic adhesive to the skin after the application of the alcoholic chlorhexidine paint, but the authors concluded that the plastic did not provide an extra benefit regarding bacterial growth or time to wound recolonisation [87].

### 4.9. Does Maintaining Adjusted Blood Glucose Levels during Surgery Reduce the Risk of Postoperative Mediastinitis?

We recommend the control of blood glucose level during surgery (preferably with continuous insulin infusion), keeping it within 110 and 180 mg/dL.

Evidence level 2+. Strong recommendation, moderate quality of evidence.

Hyperglycaemia and diabetes are known risk factors for the development of surgical site infections in patients who undergo MHS. Several studies suggest that perioperative hyperglycaemia poses an additional risk for infection among diabetics. Intra- and postoperative insulin administration protocols have been implemented.

A meta-analysis of 29 randomised trials [88] (4 of which focused on cardiac surgery) that evaluated the benefits and risks of strict glycaemic control versus usual control in critical patients in terms of mortality, pointed toward a strict control. The rate of sepsis was lower, although the number of significant hypoglycaemias was greater.

None of the randomised trials approach this question directly; evidence is based on prospective and retrospective observational studies. The most active group is that of Furnary et al. [89] in Oregon, with diabetic patients. They showed that peri- and postoperative control of blood glucose with continuous intravenous insulin infusion provides a significant benefit in terms of mortality and reduction in both superficial and deep sternal infection in comparison to intermittent control with subcutaneous insulin.

The STS Arterial Revascularisation Clinical Guidelines 2016 [90] recommend tight glycaemic control in diabetic patients undergoing revascularisation with double mammary versus single mammary to reduce the incidence of mediastinitis.

In a case–control study with diabetic and non-diabetic patients [91], the authors conclude that wound infection is more frequent in diabetics than in non-diabetics and that postoperative hyperglycaemia is more frequent in diabetics with wound infection than in non-infected diabetics, with diabetes assuming a risk factor per se, regardless of the level of glycaemic control.

Two retrospective studies analysed the outcomes in interventions performed exclusively during the postoperative period (without considering blood glucose control in the operating room). The first included more than 4600 coronary patients but it was not oriented to wound infection [92]. The second included a small number of diabetic and double mammary artery graft patients [12] that assessed infections requiring surgery, showing a benefit of blood glucose control with continuous insulin infusion during intensive care.

## 5. Diagnosis

### 5.1. Do Surveillance Cultures Performed at the Time of Mediastinal Wound Closure Allow Predicting the Risk of Mediastinitis and Anticipate the Aetiology?

We do not recommend the systematic collection of surveillance cultures at the time of closure of the mediastinal.

Evidence level 2+. Strong recommendation, moderate quality of evidence

As with other surgical wound infections (SWIs), one of the accepted theories regarding the pathogenesis of postoperative mediastinitis is that most causative microorganisms are acquired in the operating room, while wounds and tissues are exposed to the surrounding environment, and less frequently by haematogenous spreading [93]. Thus, culture samples collected from the sternum or mediastinum wound immediately prior to wound closure should show the presence of microorganisms causing mediastinitis at a later time.

In a prospective study, Bouza et al. [94] obtained several cultures of mediastinal wounds from 227 patients before wound closure at the end of the MHS in a population without signs of mediastinal infection. Overall, 31% of the patients had one or more positive cultures and 110 different microorganisms were isolated. Seven of the 227 patients developed mediastinitis, although positive surveillance cultures did not predict the risk of mediastinitis, nor was there any relationship between the microorganisms present during wound closure and those causing mediastinitis.

### 5.2. Is Radiologically Guided Needle Aspiration Convenient in Patients from Whom Parasternal or Retrosternal Purulent Collection Is Performed?

We recommend CT-guided puncture in patients whom retrosternal sample collection is performed and when there are no other means to confirm the aetiological diagnosis.

Evidence level 3. Strong recommendation, low quality of evidence.

Retrosternal aspiration may be useful in patients with suspected mediastinitis and with postoperative sepsis, particularly in the absence of local signs of sternotomy infection (inflammation, exudate and/or sternal instability). In 1984, Sarr et al. performed blind subxiphoid mediastinal aspiration in patients with fever and leucocytosis after MHS in cases without drainage or sternal instability. Early diagnosis was possible in 9 out of 24 punctured patients [95]. Benlolo et al., in a series of 1024 patients who underwent sternotomy for MHS, performed sternal puncture in a subgroup of 49 patients suspected of mediastinitis [96]. The negative predictive value in the few published experiences is very high. However, coagulase-negative *Staphylococcus* (CNS) and Propionibacterium spp. isolates may indicate skin contamination. Computed tomography (CT)-guided puncture is useful in patients to whom mediastinal collection of samples is carried out when there is suspicion of infection. Being an invasive procedure, it is not free of adverse effects and, therefore, should ideally be performed by an expert.

### 5.3. What Interpretation Should Be Given to Cultures Derived from Superficial Wounds or Fistulous Tracts in Cases of Suspected Mediastinitis?

We recommend that cultures from sites that do not represent normally sterile tissues or fluids should be interpreted with caution, since they do not always allow determination of the causative agent of mediastinitis. The identification of the microorganism and its repeated isolation along with the clinical findings might be useful for the interpretation of the results.

Evidence level 3. Strong recommendation, low quality of evidence.

The list of criteria for surgical site infections used by the National Nosocomial Infection Surveillance System (Centres for Disease Control (CDC)) includes signs and symptoms that can be directly observed by the surgeon, such as wound dehiscence or fistulous tracts. The system also offers the possibility of using a microbiological criterion, e.g., presence of microorganisms isolated from fluids or tissue cultures aseptically obtained from the organ or space. However, there are no specific requirements for cultures, species identification, strain typing and interpretation of the bacteriological findings.

In many PSM studies, a positive culture is required in addition to observable signs and symptoms. This requirement may serve to limit the accuracy of the diagnosis, since even in cases with undoubted and visible signs of infection, cultures may result negative due to the antibiotics administered to the patient before or after the surgery.

Most post-sternotomy mediastinitis series present grouped microbiological isolates of blood cultures, wound, drainage and surgical samples, and few series provide a correlation between the various types of samples [33,97,98]. It is admitted that repeated isolation of the same microorganism from wounds or fistulae, particularly in the case of S. aureus or Gram-negative bacilli, has a high PSM aetiological predictive value [99].

### 5.4. What Is the Value of Anticipating the Diagnosis of Mediastinitis from Routine Cultures of Pacemaker Wires?

We do not recommend the systematic epicardial pacing wire cultures for early diagnosis of mediastinitis in the absence of clinical signs of infection.

Evidence level 2+. Strong recommendation, moderate quality of evidence.

A few procedures to obtain samples of the anterior mediastinum have been described in the literature. These include retrosternal aspiration through sternotomy and subxiphoid retrosternal aspiration. However, the main risks of these two methods are lesions of the epicardial vessels, vascular grafts or the heart wall. Due to their location, epicardial pacemaker wires can be considered a good sample of the anterior mediastinum. In a study by Maroto et al. [100], epicardial pacemaker wire cultures were performed from 565 patients who underwent cardiac surgery with extracorporeal circulation. Cables were removed on the seventh to ninth postoperative day under sterile conditions and were grown using routine techniques for the culture of venous catheters. Mediastinitis developed in 16 patients and *S. aureus* was the most common pathogen (81.25%). The authors identified 458 true-negative, 12 true-positive, 91 false-positive and 4 false-negative results. Therefore, cultures of epicardial pacing wires for the diagnosis of mediastinitis have a sensitivity of 75%, specificity of 83.4%, positive predictive value of 11.6% and negative predictive value of 99.1%.

Even though the literature is scarce, some authors state that cultures of pacemaker electrodes removed between the seventh and ninth postoperative day may be useful for diagnosis, although a high percentage of false-positive results have been reported [101,102,103]. A positive culture of epicardial pacing wires does not appear to be a useful tool for early diagnosis of mediastinitis in the absence of clinical signs of infection.

### 5.5. Does the Information Regarding Any Microorganism Identified from Samples Not Necessarily Sterile, Cultures Grown during Patient’s Progress and Samples Different from the Original One Have Any Value?

We recommend that the interpretation of bacterial culture results other than those from the original samples, surfaces or non-sterile tissue monitoring samples must be carried out individually. Their potential significance will depend on the type of microorganism, the collection site and the clinical picture.

Evidence level 3. Strong recommendation, low quality of evidence.

In a work by Chan et al. [104], the authors describe the progression of secondary wound infections, defined by the presence of local inflammatory signs compatible with infection. Moreover, new deep tissue organism(s) not present in the initial debridement material were identified in the cultures. This sequence of events was common (31%) and resulted in prolonged hospital stays. There was an increased occurrence of polymicrobial infections, which included Gram-negative bacilli (GNB) and *Candida* spp. In addition, the authors identified several risk factors for secondary infection, such as the need of more than one revision surgery and closure of the sternum by muscle flap. It is worth mentioning the findings of Rodriguez Cetina et al. [105], who showed that even though 119 (75%) patients had positive microbiological results at wound closure, reinfection rates during readmission after wound closure showed no statistically significant differences.

Identification of new microbial isolates throughout the progression of patients who underwent sternotomy cleaning and debridement surgery must be interpreted individually. The microbiology of infected sternotomies that failed the first medical-surgical approach is frequently associated with polymicrobial infections [106]: methicillin-resistant *S. aureus*, Gram-negative bacilli, *Enterococcus* and *Candida* spp., resembling tertiary peritonitis and thus requiring more complex therapeutic strategies. On the other hand, in patients who undergo treatment with NPWT or other delayed closure modalities, it is not essential for wound cultures to be negative prior to sternotomy closure.

### 5.6. What Is the Significance of Positive Blood Cultures in Patients with Suspected Mediastinitis?

We recommend considering the presence of significant bacteraemia with no other clear origin in the 90 days after surgery as potentially indicative of mediastinitis, particularly when the isolate is *S. aureus*.

Evidence level 2+. Strong recommendation, low quality of evidence.

Although the diagnosis is difficult to predict clinically, some researchers have suggested that the presence of bacteraemia is highly suggestive of mediastinitis [97]. Fowler et al. [98] evaluated the clinical utility of blood cultures as a diagnostic tool to identify patients with mediastinitis. More recently, Nakamura et al. [107] retrospectively assessed the use of a microbiological evaluation protocol in 112 patients in the first 90 days following cardiac surgery. Microbiological evaluation of febrile patients consisted of collecting two blood samples for culture, a sample of urine, sputum and faeces on two consecutive days. The prevalence of blood cultures positive for *S. aureus* was significantly higher in patients with sternal wound infection than in patients without infection, although this difference was not observed for other microorganisms. Interestingly, there were significantly more patients with continuous bacteraemia (positive blood cultures for at least two days) in the sternal wound infection group than in the group without sternal infection. Although the data from the study by Nakamura et al. are similar to those of other authors, it should be considered that it was a retrospective study, limited to a single centre, the sample size was small, and the authors did not include afebrile patients. Even though data in the literature are limited, it seems evident that in patients with febrile processes throughout the 90 days post-MHS, positive blood cultures have a high positive predictive value, particularly in cases of infection by *S. aureus*, but not by other microorganisms. The result is particularly significant for blood cultures grown in the second or third postoperative week.

### 5.7. What Is the Value of Molecular and Other Non-Culture-Based Methods in the Diagnosis of Mediastinitis?

We do not recommend the routine use of non-culture-based methods for the aetiological diagnosis of mediastinitis.

Evidence level 3. Strong recommendation, low quality of evidence.

Molecular methods may be considered in patients with mediastinitis and previous negative cultures or those receiving antimicrobials at the time of the intervention and deep sampling.

Evidence level 3. Strong recommendation, low quality of evidence.

There are currently two types of molecular techniques. The first are those that focus on positive cultures generally requiring days or a growth phase of 4 to 8 h followed by DNA extraction, purification and PCR-based amplification. A second group of techniques consists of those directly applied to the samples obtained from the patient, allowing faster results.

Both types of techniques shorten the time of pathogen identification. In addition, they are attractive alternatives when conventional microbiological techniques fail to establish the microorganisms, particularly those that are slow growing, demanding, non-culturable, or when antimicrobial therapy may produce false-negative culture results. Rampini et al. highlighted the importance of 16S RNA PCR, which allows a pathogen to be identified in 42.9% of culture-negative samples in patients with evidence of infection [108]. However, its high cost, the great variability in the correlation rate reported by some researchers, and the few studies conducted in patients with mediastinitis prevent recommendation of widespread use [108,109,110].

### 5.8. What Aetiology-Related Determinations Are Possible in Patients with Conventional Negative Bacterial Cultures?

We recommend that for the diagnostic approach in negative culture cases, determinations should include: specific serological tests (*Brucella*, *Coxiella* and *Bartonella*), deep mediastinal samples for 16S and 18S (panbacterial and panfungal, respectively) PCRs and cultures in special media for *Mycoplasma* spp., *Ureaplasma* spp., *Legionella* spp., *Nocardia* spp., Fungi and Mycobacteria.

Evidence level 3. Strong recommendation, low quality of evidence.

Patients in whom the causative agent of mediastinitis cannot be identified by conventional culture methods present frequent diagnostic and therapeutic dilemmas. Although the main cause may be the administration of antibacterial agents at the time of sample collection, it is mandatory to consider the presence of other fastidious or slow-growing microorganisms requiring specific culture media. Besides contemplating the local epidemiology for specific serological tests, deep mediastinal samples for 16S and 18S (panbacterial and panfungal, respectively) PCRs and cultures in special media for *Mycoplasma* spp., *Ureaplasma* spp., *Legionella* spp., *Nocardia* spp., mycobacteria and fungi are recommended for patients with mediastinitis and negative cultures. A serum sample is also recommended. Mycobacterial cultures remain the essential investigation for all sample types: blood, tissue and bone biopsy, pus, and urine.

With regard to *M. chimaera*, despite being a very uncommon pathology, the European Centre for Disease Prevention [32] and the American Center for Disease Control and Prevention [33] have formulated a case definition for *M. chimaera* infections associated with open heart surgery based on three criteria: (i) any of the clinical criteria, including prosthetic valve or vascular infection, localised infection, and disseminated infection; (ii) exposure criteria, e.g., having undergone surgery requiring cardiopulmonary bypass in the 5 years prior to the onset of symptoms of infection; (iii) microbiological criteria, e.g., *M. chimaera* detected by culture or identified by DNA sequencing in an invasive sample.

Culture of *M. chimaera* from peripheral blood is the most common method of microbiological diagnosis [29]. Its sensitivity increases by performing multiple samples: three sets of mycobacterial blood cultures on different days.

### 5.9. Imaging Tests in the Diagnosis of Post-Surgical Mediastinitis

#### 5.9.1. What Is the Diagnostic Value of a Plain X-ray for the Diagnosis of Mediastinitis?

Plain X-rays are of limited use for the diagnosis of mediastinitis. We do not recommend their use as the first-choice diagnostic imaging test.

Evidence level 2+. Strong recommendation, moderate quality of evidence.

A plain radiograph has very limited use for the diagnosis of PSM. Mediastinal widening, usually due to postoperative haemorrhage and oedema, is often difficult to distinguish from the mediastinal widening in mediastinitis [111,112,113,114,115,116]. However, chest X-rays may be useful to identify and follow up other frequent changes in patients with a history of median sternotomy such as pleural effusion, laminar atelectasis or rib fractures that may cause immediate postoperative pain [111,116].

The presence of a displacement, rotation or rupture of suture wires or a widening of the sternal midline greater than 3 mm in a chest X-ray are very frequent findings in patients with dehiscence and their observation should make it suspect [117,118,119].

#### 5.9.2. What Is the Diagnostic Value of a Computed Tomography Scan?

We recommend performing a CT scan in the following cases:

- As a first-choice diagnostic imaging technique in post-surgical mediastinitis preferably at week 2 after the surgery, when gas or normal collections of the immediate post-surgery period are potentially not present.
- In patients with fever and leucocytosis without signs of infection or sternal wound drainage.
- In patients with wound infection, to establish the extent of the infection. For sternal wound assessment in patients with suspected dehiscence (multiplanar reconstructions).
- As a guide for sampling.

Evidence level 2+. Strong recommendation, moderate evidence quality.

To date, CT scans are the most widely used imaging technique for assessing patients with suspected mediastinitis. It has a sensitivity of 25 to 100% (in most series above 67%) and a specificity of 33 to 100% [114,120,121,122,123,124,125]. The best results are obtained from day 14 onwards (third week), when normal findings in the immediate postoperative period (soft tissue oedema, hematomas or free air) should be less evident [111,115,123,124,125,126,127].

CT scans are also useful for determining the extent of the infection (presternal, sternal or retrosternal) [111,113,114,115,116,126,127,128,129], particularly since the introduction of multidetector technology with the possibility of reconstruction in multiple planes [128,130,131]. The in-depth location of lesions is of great help for treatment planning [114,126].

A separation of the sternal fragments > 4 mm or a separation in successive studies on a CT scan suggests dehiscence. Multiplane reconstructions and volume rendering may also provide information on the location of the sternotomy line (median or paramedian) and the wires. A paramedian incision, and the displacement, rotation, or rupture of wires, are frequent findings in patients with sternal dehiscence [113,117,126].

The presence of osteomyelitis may go unnoticed in initial periods in a CT scan; although, in advanced phases, it is possible to detect it [114,117].

Overall, we recommend the use of CT scans in the following cases:

1. In patients with fever and leucocytosis without signs of infection or sternal wound drainage. In these patients, a CT scan may allow a diagnosis of mediastinitis or an alternative diagnosis to be established [111]. As reported in various articles, the efficacy of a CT scan in the diagnosis of mediastinitis increases by week 3, when immediate postoperative findings may simulate a retrosternal infection (e.g., oedema and erasure of soft tissues, haematomas or gas) and are no longer as evident [111,115,123,124,125,126,127].
2. Patients with wound infection, in order to establish the extent of the infection. In these cases, it is possible to differentiate between skin wound, pre-sternal or deep infection. The precise location of the lesions and their extent are of great help when planning surgical treatment [111,113,114,115,125,126,127,128,129].
3. Evaluation of sternal suture in patients with suspected dehiscence. The axial image and the reconstructions in multiple planes/3D allow the precise assessment of the degree of separation of the sternal fragments, existence or not of finishing, location of the incision (median or paramedian), condition of the wires, presence or absence of transverse fractures, etc. This information is of great interest when considering the treatment [113,117,126]. In patients with suspected sternal osteomyelitis, a complementary study with scintigraphy may be helpful [114,117,132,133,134].
4. In specific cases, as a guide for sampling. Several works (although none specifically, except that of Benlolo et al.) address the usefulness of CT scans as guides for sampling [121,126]. According to the reviewers, sampling with CT scans is a simple technique and, in experienced hands, practically free of complications.

#### 5.9.3. What Is the Indication to Perform an MRI, a Nuclear Imaging Test or a PET-CT in Patients with Suspected Mediastinitis?

We do not recommend the routine use of MRI, as there are few available data and wires can cause artefacts.

Evidence level 3. Strong recommendation, low quality of evidence.

Nuclear medicine techniques may be useful in the evaluation of sternal osteomyelitis.

Evidence level 2+. Strong recommendation, moderate quality of evidence.

There is not enough evidence to recommend the routine use of PET-CT in patients with suspected mediastinitis. However, it may be useful in cases with suspected chronic infection, as well as for monitoring response to treatment.

Evidence level 3. Strong recommendation, low quality of evidence.

Magnetic resonance imaging (MRI) is a diagnostic test rarely used in patients with suspected mediastinitis. Wires used in sternal suture may cause artefacts, hindering the assessment of the mediastinum [107,127,131].

Nuclear medicine studies with Tc-99 m HMPAO-labelled leukocytes [133,135,136,137,138,139,140], Ga-67 [134,137,141], indium 111-WBC [128,138,139], combined Tc-99 MDP and In-111 WBC [132,142,143] (combined Tc-99 MDP and In-111 WBC [144], 99mtc-labeled monoclonal granulocyte antibody scintigraphy [145] and, more recently, 99m Tc-UBI 29-41 [146], have been used in the study of post-sternotomy infection and post-sternotomy osteomyelitis [126,127,131,133,144]. Rouzet et al. [135] showed the usefulness of serial studies with planar scintigraphy in cases of suspected PSM relapse.

Recently published evidence supports the use of positron emission tomography-computed tomography (PET-CT) as a useful tool for the diagnosis and follow-up of infections associated with cardiovascular infection, particularly those related to cardiac devices. Its use in the field of mediastinitis is limited. Read et al. [147] showed the adequacy of PET-CT in chronic sternal infections. According to these authors, PET-CT may be used to locate lesions as well as to monitor response to treatment.

### 5.10. Are Imaging Tests Necessary When There Is a Clear Diagnosis of Mediastinitis?

We recommend performing a CT scan whenever there are signs of infection, despite the scarce information in the literature on this topic.

Evidence level 3. Strong recommendation, low quality of evidence.

Imaging tests in patients with clear mediastinitis may be of help in surgical planning. Furthermore, it will allow evaluation of the extent of sternal infection and the degree of involvement of adjacent structures [148]. To date, there are no studies in which the usefulness of imaging tests in patients with mediastinitis and delayed sternum closure is assessed [149].

## 6. Surgical Management

Surgical management is an essential element for mediastinitis; however, there is no literature that specifies the exact best time for the surgical treatment of mediastinitis nor are there any comparative and prospective studies that would allow a clear choice of the most effective surgical technique. We understand that the general recommendations to treat deep skin and soft tissue infection are followed and, therefore, it should be performed as soon as possible, once the patient’s hemodynamic and clinical stability is achieved.

### 6.1. Does the Administration of Topical Antibiotics before Surgical Closure of the Mediastinum Decrease the Incidence of Mediastinitis?

We do not recommend the use of topical antibiotics on the surgical site prior to closure.

Evidence level 2+. Strong recommendation, with moderate quality of evidence.

Several studies show that topical antibiotics, applied directly or sprayed, ensure much higher local concentrations in the wound than systemic antibiotics, and that this high concentration persists for several hours after wound closure [150]. Topical antimicrobial prophylaxis studies on the mediastinal surface have been published over the years, notably with vancomycin [151,152] and gentamicin. In the case of gentamicin [153], disparate conclusions [154,155,156,157] were drawn in seven studies (four randomised ones). For cefazolin, a Japanese study [158] that included almost 7000 patients over a period of 19 years demonstrated a significant reduction in mediastinitis by spraying cefazolin and gentamicin at different times during surgery (opening, pericardiotomy, cardioplegia passage, interruption of extracorporeal circulation, sternal closure, subcutaneous closure). The design of the study and the long period of execution detract from the strength of the evidence.

The cost-effectiveness of the results, the possible toxicity of some drugs (aminoglycosides, vancomycin) and the classic presence of resistance selection mean that prophylactic antimicrobial use with topical antibiotics is no longer a reality for most surgical services.

### 6.2. Is There a Specific Surgical Technique That Reduces the Risk of Mediastinitis in Adult Patients Undergoing Cardiac Surgery with Median Sternotomy?

We recommend the use of surgical steel wires to close the sternum. Superiority in reducing the incidence of mediastinitis has not been shown for other evaluated alternatives.

Evidence level 1+. Strong recommendation, high quality of evidence.

There are several surgical procedures and tools (wires, cables, plates or cementation techniques) for closure, but none have been widely adopted.

- Sternum wires. They are the most commonly used material and the majority of studies compare them with other closure systems or techniques. In a multicentre randomised study with high-risk patients [159], conventional closure was compared with cerclage-reinforced closure of both sternal halves (Robicsek procedure). The authors concluded that there was no benefit regarding infection or dehiscence with cerclage. Two retrospective trials found a significant association between the use of wires and higher rates of mediastinitis: an Italian trial in which wires were compared with nitinol staples [160] and an American trial that included only 45 patients and in which the sternum was fixed with titanium plates (SternaLock™ W. Lorenz Surgical, Jacksonville, FL, USA) [161].
  Nevertheless, a randomised study showed that conventional closure with surgical steel wires is superior to polyester suture (less mild infections in valvular patients) [162]. In addition, in a retrospective versus nitinol staples (Flexigrip^®^, Praesidia SRL, Bologna, Italy) [163], there were similar results in terms of deep infection or pain in two randomised versus sternal cables (Flexigrip^®^) [164] and closure with Mersilene^®^ tape (Ethicon, Inc., Somerville, NJ, USA) (braided Dacron) [165]; and in a substudy of propensity analysis with mating of the Anglo-Australian nitinol staples [163].
- Other prevention systems. A randomised trial [166] on coronary patients with/without associated valvular surgery compared soft tissue closure (saphenectomy, sternotomy) and suture impregnated with Triclosan (bactericidal and fungicidal agent) against unimpregnated suture. No differences were found for deep infections. A review of the literature on the postoperative use of preventive NPWT [167] that included three heterogeneous studies with evidence level 2 or 3 concluded that NPWT may be recommended in populations at higher risk of developing mediastinitis.

### 6.3. What Is the Prophylactic Value of Negative Pressure Wound Therapy to Avoid Mediastinitis?

In high-risk patients, we recommend the use of prophylactic negative pressure wound therapy to reduce the incidence of infection.

Evidence level 2+. Strong recommendation, moderate quality of evidence.

Good correlation has been reported between universal surgical risk estimation systems and the incidence of surgical wound infection [168]. A study on deep sternal wound infections or mediastinitis showed that more than two-thirds of the infections had the same aetiology: dehiscence of the skin suture, particularly in obese patients. Only a small proportion was caused by perioperative contamination. In most infections, the key element appears to be the dehiscence of the skin suture and the subsequent penetration of cutaneous microbiota into the sternum [169].

Prophylactic closed NPWT, e.g., Prevena (KCI) or PICO (Smith and Nephew), help keep wound edges together to avoid dehiscence, reduce lateral tension and oedema, increase tissue perfusion, stimulate granulation tissue formation, reduce bacterial colonisation and isolate the wound from potential contaminating sources. In one prospective study [170,171] with obese patients, the use of NPWT was compared prophylactically on clean incisions. A second study [165] included more than 200 patients with sternotomy. Both studies concluded that NPWT reduces the high rate of infections presented by high-risk obese patients from 16% to 4% and from 3.4% to 1.3%, respectively, in subjects with different risk factors.

Besides the two consensus conferences in which the use of NPWT was strongly advised, there is a volume of evidence for cases presenting one or more high risk factors or in subjects undergoing cardiac or pulmonary transplantation [172,173]. This evidence comes from several well-designed randomised prospective studies (clinical trials) that may be extrapolated to cardiac surgery because clean procedures were performed by orthopaedic surgeons. There is a general consensus regarding the benefits of using NPWT in patients with high risk of infection [173,174].

Complications related to the surgical treatment of DSWI are usually minor; however, life-threatening bleeding can occur. These are usually due to the rupture of the right ventricle (RV) that has been reported both following conventional treatment and negative pressure wound therapy. However, this risk can be decreased by ensuring sternal stability and possibly by releasing retrosternal adhesions after wire removal; it is also advisable that an experienced surgeon should perform NPWT revisions in an operating room.

### 6.4. During the Postoperative Period, Is There Evidence That Sternal Immobilisation Systems Reduce the Risk of Mediastinitis Compared to Conventional Bandages?

We recommend the use of postoperative sternal immobilisation systems in all patients who undergo major cardiac surgery.

Evidence level 2+. Strong recommendation, moderate quality of evidence.

Postoperative sternal instability due to dehiscence or infection is a serious complication that may result in increased morbidity or hospital stay, the need for reintervention and greater cost. It may also increase mortality to as much as 25%. In this scenario, it is important to establish preventive strategies and additional postoperative measures, including the use of thoracic immobilisation systems.

Prospective randomised studies have been conducted to assess the effectiveness of these immobilisation systems, as well as their ability to prevent sternal complications during the postoperative period after cardiac surgery.

The vest [175] consists of two longitudinal pads placed by compressing both sides of the sternum using a custom-made anterior and posterior stabilisation cerclage system to prevent intrinsic movement of the sternum boards during coughing, deep breathing, and night-time movement. Its use is somewhat annoying and requires the collaboration of the patient. However, a randomised prospective multicentre study with over 1500 patients [176] concluded that the group of patients who used the vest had a lower cumulative rate of complications (0.61% vs. 3.87% *p* = 0.047), such as dehiscence (0% vs. 0.77%, *p* = 0.046), deep infection (0% vs. 1.99%) and superficial infection (0.6% vs. 1.1% *p* = 0.417). In addition, hospitalisation time for complications was significantly shorter in the vest group (14.7 ± 707) compared to the control group (17.3 ± 17.5, *p* = 0.04). These findings have been validated in two randomised prospective multicentre studies, in which routine use of the vest implied significant prevention of sternal dehiscence and decreased the relative risk of complications from deep infection [177,178].

### 6.5. Should Mediastinitis Patients Be Treated with Mediastinal Lavage? For How Long?

We do not recommend mediastinal lavage on a routine basis, except in patients in whom NPWT cannot be performed or who require immediate closure. Povidone iodine should not be used in any case.

Evidence level 2+. Strong recommendation, low quality of evidence.

Conventional treatment of patients with mediastinitis after median sternotomy usually includes surgical revision, closure with mediastinal lavage, or reconstruction with omentoplasty or pectoralis plasty, and more recently, treatment with NPWT [179,180,181,182]. Mediastinal lavages are normally performed by inserting two irrigation catheters in combination with two or three mediastinal aspiration drains, placed after surgical cleaning, tissue debridement, and sternal resuture in the operating room [179]. Merrill et al. [183] report good results with this technique in 40 patients treated consecutively for mediastinitis, concluding that surgical debridement, sternal closure and mediastinal lavage are an effective treatment and an appropriate option for the management of patients with mediastinitis. Molina et al. [184] also report good results in their series of 114 patients with mediastinal lavages. Deschka et al. [185] compare the results of surgical cleaning, sternal closure and mediastinal lavages versus isolated sternal cleaning and resuture, reporting better results in the group of subjects who received mediastinal lavages.

However, NPWT provides new possibilities. Several articles [180,181,182,186] suggest that NPWT offers better results with their increasingly widespread use in many centres, higher cure rates, lower infection recurrence rates, and shorter stays in ICUs and hospital wards. However, in most of the reported series, no differences were found in terms of mortality.

In patients who have no other choice but to undergo a mediastinal lavage, it should not be maintained beyond one week. Moreover, negative drainage cultures should not be used as criteria for withdrawing or maintaining the tubes [183,187]. In no case is the use of continuous lavage with PI indicated, due to renal, metabolic or thyroid function toxicity [184,188].

### 6.6. What Is the Best Surgical Reconstruction Technique?

We recommend choosing the surgical reconstruction technique according to the stage, sternal stability and bone viability.

Evidence level 2+. Strong recommendation, low quality of evidence.

We did not find any randomised studies comparing surgical sternal reconstruction techniques. Most of the works are observational, cohort, case series and isolated case reports. Two meta-analyses support the use of NPWT during the initial management of sternal wound infections [189,190]. In most of the published studies, the lack of use of standardised severity scales makes it difficult to compare results and recommend the best surgical technique.

There are numerous classifications of mediastinitis: Pairolero and Arnold [191], Oakley and Wright [1], Jones et al. [192], Greig et al. [193], Grädlund et al. [169], and Windergen et al. [194]. We recommend the use of the Windergen et al. classification due to its easy implementation, therapeutic approach and the recent incorporation of NPWT.

#### 6.6.1. Wound with Minimal Bone Loss, Relatively Stable Sternum

Current evidence supports the use of NPWT as a first-line treatment or bridge for surgical closure (IB) [182,189,190,195,196,197,198,199,200,201,202,203,204,205,206,207,208,209]. Early diagnosis and rapid application of NPWT improve the assessment of infection [210] and surgical closure outcomes, and decrease the risk of sepsis and the occurrence of infection-related complications [192,210,211,212,213,214,215]. In hemodynamically stable patients and in cases where NPWT is not available, direct wound debridement closure and direct closure with muscle flap reconstruction are widely accepted options [216,217,218].

#### 6.6.2. Unstable Sternum and Viable Bone Wounds

Recent studies suggest that the initial application of NPWT followed by sternal rewiring or plates and coverage by muscle flaps improves the results of sternal reconstruction; the use of NPWT is recommended over continuous irrigation systems [181].

After sternal stabilisation, coverage with bilateral pectoral muscle advancement flaps [219,220,221,222] or omentum flap increases the chances of success [223,224].

#### 6.6.3. Unstable Sternum and Non-Viable Bone

In cases of significant sternal destruction, muscle flap coverage is necessary to provide stability to the chest and improve wound vascularisation [225,226]. Different techniques may be used: bilateral pectoralis flaps (advanced or rotational), the rectus abdominis flap, the dorsalis flap or omental flaps (omentoplasty) [227].

Omental flaps have angiogenic properties, are a source of granulation tissue and are more effective against infection [228,229,230,231]. Thus, omental flaps are recommended in the presence of resistant microorganisms (SAMR, *Candida*), in diabetic patients or the visualisation of prosthetic material [208,232,233,234,235,236,237,238,239].

### 6.7. What Is the Risk of Developing Sternal Dehiscence and Mediastinitis with the Use Bilateral Harvesting of Internal Mammary/Thoracic Arteries as Grafts?

We recommend using BITA grafts in low-risk patients.

We recommend skeletonised BITA grafting in diabetic patients with multivessel CAD.

If there are any other related risk factors, the use of BITA must be individualised, taking into account the risk vs. benefit of the procedure. When its benefit is not clear, the use of BITA should be avoided.

Evidence level 3. Strong recommendation, low quality of evidence.

The use of bilateral internal thoracic arteries (BITA) grafting during myocardial revascularisation reportedly provides a survival benefit over single internal thoracic artery (SITA) grafting [240].

However, the use of BITA may play a role in the development of DSWI because the vascularisation of both sides of the sternal wound is compromised. Techniques aiming to preserve a better sternal vascularisation such as skeletonisation or the use of harmonic scalpel have developed over the years [241].

Factors such as obesity, COPD, female gender, old age, diabetes mellitus, renal failure and peripheral vascular disease may play a role in the development of DSWI [242] when BITA grafts are performed. For this reason, BITA grafts remain underused [243,244].

A review of the literature on this topic shows that BITA grafts do not seem to increase the rate of DSWI in patients at low risk for mediastinitis.

As reported by De Paulis (2005), in diabetic patients, as a single risk factor, BITA grafts do not seem to increase the rate of DSWI if both mammary arteries are used skeletonised rather than pediculated [245]. When other risk factors are present, especially COPD, female gender and obesity, different studies offer much more controversy. Raza et al. suggest avoiding the use of BITA in obese female diabetic patients [246]. Puskas recommends avoiding the use of BITA in morbidly obese diabetic females with high values of HbA1c [247]. Lev Ran et al. (2003) suggest avoiding the use of BITA in obese patients, associated with COPD and emergency surgery [248].

### 6.8. Are Negative Cultures Necessary before the Definitive Sternal Reconstruction? What Is the Incidence and Risk Factors of Therapeutic Failure and Recurrence?

We do not recommend delaying surgical closure based on the persistence of positive cultures.

Evidence level 3. Strong recommendation, low quality of evidence.

The incidence of recurrence of sternal infection after initial reconstruction has been estimated to be between 5 and 10% [219,222]. Although early application of NPWT improves treatment outcomes for mediastinitis, prolonged application of NPWT has been associated with increased late mortality [249] linked to infection recurrence and chronic infection [200,219,250]. The ideal duration of NPWT use remains unclear.

Bacteraemia, wound depth > 4 cm, degree of exposure and sternal instability have also been associated with the risk of recurrence of infection after closure [251]. Plasma C-reactive protein levels <30–70 mg/L at the time of sternal reconstruction have been linked with a lower recurrence rate of mediastinitis [204,252].

Although recent articles [105,232] suggest that obtaining negative wound cultures at closure does not affect the prognosis of reconstruction, other authors state that sternal closure with positive wound cultures increases the risk of infection recurrence [219].

### 6.9. What Is the Therapeutic Indication for NPWT? How Should Progression Be Assessed and Its Duration Be Calculated?

We recommend applying NPWT considering the following: the stability of the sternum, as a curative method (with or without surgery) in patients with stable sternum, or as a bridge technique in preparation for surgery in subjects with an unstable sternum. NPWT should be checked every two to three days and last no longer than three weeks.

Evidence level 2+. Strong recommendation, moderate quality of evidence.

Argenta and Morykwas introduced this therapy in the 1990s [253] and it began being used in Europe in 1997. A negative pressure is applied using a polyurethane sponge with a pore size ranging from 400 to 600 μm. The sponge is connected to a vacuum tube that drains into a container and is covered with an adhesive to ensure there are no leaks. The suction can be continuous or intermittent, with different intensities.

The knowledge and experience of NPWT is established and accepted. It is thought to stimulate the growth of granulation tissue [215], extract exudates and increase blood flow in the wound. The patient can be moved and receive early rehabilitation [254,255,256], which may be associated with a lower hospital cost [257]. However, most studies are small observational studies—with a limited population and short-term follow-up—on the treatment of mediastinitis and sternal dehiscence [182,212,215,257,258]. The role of bacterial count in patients in whom NPWT is applied [259,260] remains to be defined.

We recommend changing the system and carrying out visual inspection every 48 to 72 h, which can be used to obtain culture samples.

## 7. Medical Management

### 7.1. When Should Empirical Antimicrobial Treatment Be Initiated?

In adults with signs and symptoms of severe acute infection, we recommend initiating empirical antibiotic treatment as soon as there is clinical suspicion of mediastinitis.

In non-critical adults, empirical treatment can wait for targeted treatment, based on laboratory findings.

Evidence level 3. Strong recommendation, low quality of evidence.

We did not find a high level of evidence nor high-quality studies, i.e., clinical trials, cohort or case–control studies, on when is the best time to start empirical antimicrobial treatment. Most of the literature refers to case presentations or reviews of the topic. Two articles assess mortality in patients with mediastinitis. The recommendation in these works for acute mediastinitis is to initiate early antibiotic treatment (as soon as there is clinical suspicion of mediastinitis and after collection of samples for culture) [261,262]. In the case of non-critical situations, it is not necessary to begin treatment empirically, although it is advisable to obtain samples that allow a treatment to be indicated based on microbiological findings.

### 7.2. Should Coverage against Methicillin-Resistant Staphylococci Be Systematically Included?

In critically ill adult patients for whom aetiological confirmation is a threat, we recommend including methicillin-resistant *Staphylococcus* coverage for the empirical treatment depending on local susceptibility pattern.

Evidence level 4. Conditioned recommendation based on expert opinion. Very low level of evidence.

Evidence on treatments targeted to specific microorganisms in patients who develop mediastinitis is scarce and of poor quality. It is limited to retrospective studies or case series with a small number of patients [263,264,265,266]. The recommendations in this document are based on expert opinion, and local epidemiology should always be considered before treatment initiation.

Mediastinitis secondary to cardiothoracic surgery is mainly caused by Gram-positive cocci, with *staphylococci* causing the infections in more than 60% of the cases [267,268]. Morisaki et al. [206] and Karra et al. [261] showed that infection by MRSA was the factor most strongly associated with increased in-hospital mortality and with one-year mortality, respectively.

We must not forget that in addition to *S. aureus*, coagulase-negative *staphylococci* (CNS) are a very frequent cause of mediastinitis and that these microorganisms are usually resistant to methicillin everywhere. Therefore, it seems reasonable that empirical treatment should offer coverage against methicillin-resistant *staphylococci*, not only to cover *S. aureus* but also coagulase-negative *staphylococci* (Table 3).

### 7.3. When Should Empirical Coverage against Gram-Negative Bacilli Be Included?

In the empirical treatment of adult patients with acute mediastinitis, we recommend including coverage against Gram-negative bacilli, considering local epidemiology (Table 3).

Evidence level 2−. Strong recommendation. Low level of evidence.

Several series show that the proportion of cases of mediastinitis caused by GNB ranges between 15 and 25% [269,270]. In addition, infection with these microorganisms was associated with a poorer prognosis in subjects with mediastinitis. Charbonneau et al. [269] showed that in-hospital mortality at 30 days was significantly higher in patients with GNB mediastinitis compared to those caused by Gram-positive bacteria (31.9% versus 17.0%; *p* = 0.004) [269,271].

In a recent study published by Ma et al. in 170 patients [270], 87 GNB were isolated, the most common being *P. aeruginosa* (*n* = 40, 25.5%), followed by *A. baumannii* (*n* = 25, 15.9%), *Enterobacter cloacae* (*n* = 15, 9.6%), and other Gram-negative pathogens (*n* = 7, 4.5%).

In addition to the above indicated figures, in recent years, there has been a high proliferation of GNBs that deserve to be described as multidrug resistant (MDR) and which lead to a worse prognosis.

### 7.4. When Should Empirical Coverage against Fungi Be Included?

Overall, antifungal treatment should not be systematically included as part of the empirical treatment of PSM. It should only be administered in critical situations where there are risk factors for invasive fungal infection.

Evidence level 4. Conditioned recommendation based on expert opinion. Very low level of evidence.

The proportion of patients with postoperative mediastinitis of fungal aetiology is below 5%. In addition, the isolation of yeasts or filamentous fungi in a torpid course surgical wound is very difficult to distinguish from colonisation [272].

We recommend that indications for empirical antifungal treatment be reduced exclusively in critically ill patients who have predisposing factors of invasive fungal infection, irrespective of the fact of having mediastinitis

### 7.5. Is Topical Use of Antimicrobial Agents Beneficial?

With the current available information, it is not possible to recommend mediastinal irrigation either with antibiotics or antiseptic substances.

Evidence level 3. Strong recommendation, low quality of evidence.

Although topical irrigation of antimicrobial agents is a widely used practice by surgeons of different specialties, their use is uncontrolled and there are no studies in the literature with sufficient evidence to support or contradict their use.

The purpose of mediastinal antibiotic irrigation is to achieve a very high local concentration (there is evidence that mediastinal gentamicin levels may reach levels high enough to be effective against resistant microorganisms) with low serum concentrations. However, there are no data on the effect that a continuous irrigation system has due to bathing the surgical site with high doses of antimicrobial solutions or the irrigation technique itself, which may have the advantage of washing out large amounts of fibrin, clots and detritus from the infected area. It is also unknown how much antimicrobial is absorbed through the mediastinum or pleural cavity, with the possibility of systemic side effects or resistance building.

#### 7.5.1. Topical Use of Antibiotics

In 1963, Shumacker and Mandelbaum introduced for the first time a system of retrosternal lavage with antibiotics in patients with mediastinitis [273]. Neomycin irrigation was used for years and, ultimately, removed due to toxicity. In the case of gentamicin, the degree of absorption was evaluated after continuous irrigation in patients with non-infectious sternal dehiscence. Toxic plasma levels were found, as well as insufficient/inadequate effects, depending on variables such as body surface and gender. When using gentamicin, it is recommended to motorise its concentration during mediastinal irrigation [274]. Leyh et al. [275], in a series of 42 patients with mediastinitis, treated with gentamicin implants (Collatamp® Schering-Plough, Stockholm, Sweden), observed high bactericidal levels in the mediastinal fluid with low plasma concentrations.

Other studies that use drugs to treat Gram-positive bacteria are usually retrospective, include a low number of cases and many factors that make interpretation difficult. Some only use animal models [276].

#### 7.5.2. Topical Use of Other Antimicrobials

The use of iodine povidone (IP) in irrigation was referred to as effective in the treatment of mediastinitis until the 1980s [277]. Subsequent studies warn of possible local and systemic toxicity. In 1985, Glick et al. described significant iodine absorption in a 34-month-old infant, resulting in metabolic acidosis and death [278]. The same authors observed a linear absorption of iodine through the mediastinum associated with the concentration and rhythm of infusion (similar to an intravenous infusion) in an animal model [279]. New cases of toxicity with renal failure and subsequent seizures have been reported [280,281]. Thus, its use is contraindicated.

In a study by Truillet et al. of 19 cases, the authors describe the results of coverage of mediastinum with sugar, with rapid formation of granulation tissue and no evidence of side effects [282]. Szerafin et al. and De Feo et al. (both series of nine patients) reported similar results with the same technique [283,284]. This natural agent has ceased to be used in many centres with the recent introduction of NPWT systems.

Gentian violet seems to have replaced PI as an antiseptic in mediastinal irrigation due to its low toxicity and excellent anti-staphylococcal activity (including methicillin-resistant *S. aureus*) and against Gram-negative bacteria. The only published experiences on this are isolated case communications [285,286,287].

### 7.6. What Are the Indications and When to Switch to Oral Antimicrobial Agents?

We recommend the use of sequential antimicrobial treatment in stable patients who have received adequate surgical treatment after a period of i.v. therapy, the duration of which is difficult to determine. Active antimicrobials with high bioavailability should been used, depending on the aetiology.

Evidence level 2−. Strong recommendation. Low level of evidence.

The available information is scarce and based mainly on descriptive studies with a limited number of cases from a single centre. These studies mostly describe cases whose aetiology is predominantly staphylococcal and were initially treated with i.v. antimicrobials for two to three weeks and continued with oral antimicrobials for several weeks [288]. Antibiotics with high bioavailability such as fluoroquinolones (levofloxacin 500 mg/12 h or ciprofloxacin 500–750 mg/12 h), cotrimoxazole 160/800 mg/12 h or clindamycin 450 mg/8 h were used, in many cases associated with rifampicin. One of the studies showed that the association of rifampicin with older drugs significantly improved outcomes [289]. Another possible association would be with minocycline, 100 mg every 12 h. The experience with linezolid is more limited. In a study of cases of sternal osteomyelitis, linezolid monotherapy was used for 28 days, and was associated with a significant number of relapses, which the authors attributed to insufficient duration of the treatment. Usually, no defined criteria are established for the oral switch. No considerations are made regarding the microorganism, presence of bacteraemia or osteomyelitis. The average duration of oral treatments was around six weeks, except in cases of osteomyelitis which, in one study, lasted between 6 and 18 months [104]. Most of the patients were also treated surgically by drainage and debridement of abscesses, removal of foreign bodies, and with NPWT in more recent studies [290]. These studies do not provide enough data to allow for a comparison of results between patients treated sequentially and those who continued intravenously.

However, in recent years, the shift from i.v. treatment to oral treatment with highly bioavailable drugs of different severe infections has been shown not to be inferior to continuing i.v. treatment, with clear advantages for patients and institutions [291].

### 7.7. What Is the Antibiotic Treatment of Choice for Mediastinitis Confirmed to Be Caused by Gram-Positive Cocci, Including Multidrug Resistant Microorganisms?

We recommend the use of beta-lactam drugs such as isoxazolyl penicillins or cefazolin in patients with methicillin-sensitive staphylococcal mediastinitis.

We recommend the use of glycopeptides or glycolipopeptides (vancomycin or daptomycin) in patients with methicillin-resistant staphylococcal infections.

In enterococcal mediastinitis, particularly in bacteremic patients, it is recommended to follow the accepted scheme for endocarditis with double beta-lactam treatment (ampicillin + ceftriaxone) or the beta-lactam–aminoglycoside combination.

These treatments always require expert consultation.

Evidence level 3. Strong recommendation, low quality of evidence.

Gram-positive bacteria are the most frequent cause of mediastinitis and, in decreasing order, the most frequent are: coagulase-negative *Staphylococcus* (CNS), MSSA, MRSA and *Enterococcus* spp. [266].

We have not found any clinical trial with any drugs specifically performed in patients with mediastinitis. Therefore, recommendations regarding the indication of drugs and the duration of treatment should be interpreted with caution and derive from experience in other fields such as infectious endocarditis [292], infection of skin and soft tissues of organs or spaces, and bacteraemia [98], many of which are seen in patients with mediastinitis.

In patients with MSSA, beta-lactams are the drugs of choice, particularly isoxazolyl penicillins (cloxacillin or oxacillin) or first-generation cephalosporins (cefazolin). According to recent studies, there is no evidence that either group of drugs is superior to the other in patients with bacteraemia and infective endocarditis [293]. Cefazolin allows a more comfortable administration (three daily doses) in comparison to cloxacillin (six daily doses). We found no evidence implying a need to associate drugs with the first-choice agents described above. Glycopeptides would be reserved for patients with beta-lactam intolerance (Table 4).

If the causative agent is MRSA or CNS, where methicillin-resistance usually exceeds 50% of cases, the use of vancomycin or daptomycin is recommended as a first choice. At present, it seems sensible to use these first-choice drugs combining daptomycin with a second agent (e.g., cloxacillin, ceftaroline, fosfomycin) for patients with MRSA isolates given the results in the field of bacteraemia. The most commonly recommended combinations are vancomycin/daptomycin-ceftaroline or daptomycin/vancomycin-fosfomycin (see Table 4)

With regard to the association to rifampicin, it would be reserved for patients in which the prosthetic material could not be extracted and is supposedly infected [294].

Alternatives to the first-line drugs mentioned above may be considered in different circumstances, but we insist that, to date, there are no clinical trials carried out in patients with mediastinitis. Ceftaroline may be a suitable agent as it is a beta-lactam with activity against MRSA. Oxazolidinones, such as linezolid and tedizolid, are an alternative but have not been evaluated in treatments of the duration required for mediastinitis. Its use is often performed sequentially and to complete an i.v. treatment with beta-lactams and/or glycopeptides, of which 15 or more days of treatment with good evolution have already been performed.

The use of long half-life glycopeptides, such as dalbavancin or oritavancin, has been studied in skin and soft tissue infections but not in cases of mediastinitis. These drugs do not have an official indication for prolonged treatments as in the case of mediastinitis, although a single retrospective, observational, cohort study has shown optimistic results.

It is difficult to give firm recommendations on the use of alternative agents such as cotrimoxazole, iclaprim, tigecycline, fosfomycin, etc., which should only be indicated under the indication and follow-up of an expert.

*Enterococcus* is a less common cause of mediastinitis. In patients with beta-lactam-sensitive *Enterococcus*, as is generally the case with *E. faecalis*, we recommend the combination of ampicillin and ceftriaxone (using the accumulated data and experience in patients with endocarditis as criteria). Alternatively, the classic ampicillin and gentamicin regimen may be used, provided that resistance to gentamicin is of low profile and with an MIC < 500 ug/mL. This antibiotic combination is more nephrotoxic, and we consider it an alternative to the previous one and not as the primary recommendation. In patients with ampicillin- and vancomycin-resistant *Enterococcus*, treatment should be scheduled and followed by an expert. In some cases, the combination of daptomycin with a second drug (e.g., ampicillin, ceftaroline) has been used successfully.

Other Gram-positives are rarely causative agents of mediastinitis and their treatment should be agreed with by antibiotherapy experts.

### 7.8. What Is the Antibiotic Treatment of Choice for Mediastinitis Caused by Gram-Negative Bacilli Including Multidrug Resistant Microorganisms?

We recommend that the selection of antimicrobial treatment in patients with proven GNB mediastinitis must be adjusted in each circumstance and under expert supervision.

Evidence level 3. Strong recommendation, low quality of evidence.

Although Gram-positive microorganisms are the cause of most cases of post-sternotomy mediastinitis, some authors have reported a prevalence of Gram-negative bacilli (GNB) infection of up to 56.7% (*K. pneumoniae* 16.4%) [271].

Targeted treatment of mediastinitis caused by GNB follows the same general criteria used for other types of infections. There are three possible situations: GNB with common sensitivity patterns, extended spectrum beta-lactamases (BLEE) GNB producers, and multidrug resistant GNB. We refer the reader to the more general references for these treatments [295,296,297,298,299,300,301], but would like to highlight certain key aspects of particular relevance for mediastinitis.

We suggest using beta-lactams when possible.

The duration of the treatment is always longer than in other soft tissue infections, usually varying between four and six weeks.

In GNB mediastinitis also, it is acceptable to switch to oral treatment when drugs with adequate bioavailability and tolerance exist.

In patients with MDR microorganisms, a drug association should normally be chosen, although the availability of new agents may make this recommendation obsolete.

Given the prolonged treatment duration of these patients, it is particularly desirable to avoid the use of toxic agents such as colistin and aminoglycosides.

Given the difficulties of therapeutic choice in these circumstances, they make the contribution of the infectious disease expert even more indispensable.

### 7.9. What Is the Treatment of Choice for Mediastinitis Caused by Fungi?

We recommend confirming fungal mediastinitis before empirical treatment. The treatment described in Table 4 is only indicative and always requires expert consensus.

Evidence level 3. Strong recommendation, low quality of evidence.

Although cases of fungal mediastinitis are anecdotal, they are associated with a high mortality rate. *Candida* species are the most frequently reported microorganisms [302]. Table 4 summarises the recommended guidelines [303,304]. The reported experience for the treatment of sternal *Candida* infection is limited to a few clinical cases [305,306,307,308]. This is not entirely analogous to post-surgical sternal osteomyelitis, where the blood supply to the sternum after sternotomy has been interrupted and there is often foreign material (sternum wires, etc.), making the penetration of antimicrobial agents at the site of infection more difficult.

Malani et al. [309] suggest that treatment with azoles is usually effective, but should be administered for at least six months, or longer, if CT scans reveal bone destruction.

Amphotericin B has historically been the primary drug of treatment for *Aspergillus* infections [310]. Recent data have shown the superiority of voriconazole over amphotericin as the antifungal therapy of choice for most forms of invasive aspergillosis [311]. However, clinical experience with *Aspergillus* infection in the sternum remains limited.

### 7.10. What Criteria Make It Possible to Estimate the Duration of Treatment for Mediastinitis?

We recommend an average duration of four to six weeks in bacterial mediastinitis. When possible, we recommend switching to oral antimicrobials at week two to three.

Evidence level 4. Conditioned recommendation based on expert opinion. Very low-grade quality of evidence.

In case of sternal osteomyelitis and/or fungal mediastinitis, we recommend prolonged treatment.

Evidence level 4. Conditioned recommendation based on expert opinion. Very low-grade quality of evidence.

There are no comparative studies on the optimal duration of antibiotic treatment for patients with mediastinitis. Recommendations are based on clinical judgment, evolution of acute phase reactants, microbiological studies and imaging tests.

The duration of treatment for bacterial mediastinitis is 4–6 weeks. Initial intravenous therapy is given for two weeks. After sternum closure, and if the results of antibiotic susceptibility tests allow it, it is recommended to switch to oral antibiotics, always considering the results of the antibiogram. If sternal osteomyelitis or foreign bodies (cerclages) are present, oral treatment may be prolonged.

## Figures and Tables

**Table 1:** Levels of evidence derived from the articles reviewed.

Quality of Evidence	
1++	High quality meta-analysis, SR of a RCT or a RCT with a very low risk of bias
1+	Well-conducted meta-analysis, SR of a RCT or a RCT with a low risk of bias
1−	Meta-analyses, SR, RCT or RCT with a high risk of bias
2++	High-quality SR of case–control or cohort studies
2+	High quality or cohort case and control studies with very low risk of confusion or bias and a high probability that the relationship is causal
2−	Well-executed case–control or cohort studies with low risk of confusion or bias and a moderate probability that the relationship is causal
3	Non-analytical studies (e.g., clinical cases, case series)
4	Expert opinion(s)

Studies with a “−” level of evidence should not be used as a basis for a recommendation. Adapted from the Scottish Intercollegiate Guidelines Network. RCT = randomised control trial; SR = systematic review.

**Table 2:** Classification of recommendations.

Degree of Recommendation	Risk vs. Profit	Methodological Strength of Evidence
Strong recommendation, high quality of evidence	Benefits clearly outweigh the risk	Consistent evidence from randomised controlled trials without major limitations or exceptionally strong evidence from observational studies
Strong recommendation, moderate quality evidence	Benefits clearly outweigh the risk	Evidence from randomised controlled trials with relevant limitations (inconsistent results, methodological weaknesses, indirect or imprecise) or very strong evidence from observational studies
Strong recommendation, low or very low quality of evidence	Benefits clearly outweigh the risk	Evidence of at least one critical outcome from observational studies, case series or randomised controlled trials, with serious defects or indirect evidence
Weak recommendation, high quality of evidence	Close benefit/risk balance	Consistent evidence from randomised controlled trials without major limitations or exceptionally strong evidence from observational studies
Weak recommendation, moderate quality of evidence	Close benefit/risk balance	Evidence from randomised controlled trials with relevant limitations (inconsistent results, methodological weaknesses, indirect or imprecise) or very strong evidence from observational studies
Weak recommendation, low or very low quality of evidence	Uncertain risk/benefit estimates; possible close benefit/risk balance	Evidence of at least one critical outcome from observational studies, case series or randomised controlled trials, with serious defects or indirect evidence

**Table 3:** Recommendations for empirical antibiotic treatment.

Recommendation	Dose
* Daptomycin or vancomycin + piperacillin-tazobactam or meropenem (depending on the centre)	High doses (8–10 mg/kg/day) of i.v. daptomycin 1 g/12 h of vancomycin and later adjustment considering concentration 4.5 g/6–8 h of i.v. piperacillin/tazobactam Meropenem: 1–2 g/8 h i.v. Aztreonam 2 g/8 h i.v.
Allergy: * Daptomycin or vancomycin + meropenem or aztreonam	

* Treatments should be reviewed considering to the results of the microbiological cultures and clinical progression.

**Table 4:** Recommendations * for the treatment of mediastinitis as per the aetiological agent. Treatment must always be carried out with the advice of an expert in infectious diseases and adjusted to the sensitivity of each microorganism in each centre.

Microorganism	First Choice	Alternatives
Gram-positive cocci		
Methicillin-sensitive *Staphylococcus aureus*	Cloxacillin/cefazolin	
Methicillin-resistant *Staphylococcus aureus*	Vancomycin or daptomycin + cloxacillin/ceftaroline Allergic to BL Daptomycin + fosfomycin Daptomycin + cotrimoxazole	Ceftaroline Fosfomycin + imipenem Clindamycin + cotrimoxazole Telavancin ** Oral antimicrobials
*Enterococcus faecalis*	Not high aminoglycoside resistance Ampicillin + ceftriaxone Ampicillin + gentamicin	Vancomycin + gentamicin
	High aminoglycoside resistance Ampicillin + ceftriaxone	Daptomycin + Fosfomycin
*Enterococcus faecium*	Daptomycin + ampicillin Daptomycin + ceftaroline	Daptomycin + tigecycline Tigecycline + gentamicin
Gram-negative bacilli including multidrug resistant microorganisms		
*Enterobacteriaceae*	According to antibiogram Meropenem if BLEE	Tigecycline + amikacin or imipenem
*Pseudomonas*	According to antibiogram Piperacillin-tazobactam if susceptible	Ceftazidime + amikacin Ceftolozane-tazobactam
*Acinetobacter*	Tigecycline +/− colistin or meropenem if susceptible	
Carbapenemase-producing	Expert consensus is required Ceftazidime-avibactam if susceptible.	Expert consensus
Fungi		
*Candida*	Fluconazole/Voriconazole	Echinocandins Liposomal amphotericin
*Aspergillus*	Voriconazole	Liposomal amphotericin Echinocandins Combinations

* Before prescribing, always search for drug interactions and patient allergies. ** Oral antimicrobials (after 2–3 weeks of IV treatment and according to antibiogram): Linezolid/tedizolid, trimethoprim-sulfamethoxazole, clindamycin, quinolones, fusidic acid with/without rifampicin.

## Data Availability

Not applicable.

## Abbreviations

A1cHb	A1c blood glycosylated haemoglobin
*CIBERES*	Biomedical Research Centre Network for Respiratory Diseases
BMI	Body mass index
CDC	Centres for Disease Control
CH	Chlorhexidine
CNS	Coagulase-negative *Staphylococcus*
CT	Computed tomography
GNB	Gram-negative bacilli
IPA	Isopropyl alcohol
MRI	Magnetic resonance imaging
CS	Cardiac surgery
MRSA	Methicillin-resistant *Staphylococcus aureus*
MSSA	Methicillin-sensitive *Staphylococcus aureus*
MDR	Multidrug resistance
NPWT	Negative pressure wound therapy
PCR	Polymerase chain reaction
PICO	Population, intervention, comparison and outcome
PET-CT	Positron-emission-tomography/computed tomography
PSM	Post-surgical mediastinitis
PI	Povidone iodine
SIGN	Scottish Intercollegiate Guidelines Network
STS	Society of Thoracic Surgeons
*SEQ*	Spanish Journal of Chemotherapy
*SEICAV*	Spanish Society of Cardiovascular Infections
*SECTCV*	Spanish Society of Thoracic-Cardiovascular Surgery
SWI	Surgical wound infection

## Summary

Summary table of recommendations and level of evidence.

**Section**	**Recommendation**	**Grade of Evidence/Strength of Recommendation**
Prevention	We recommend optimising preoperative glycaemic control in diabetic patients with high HbA1c levels (>6.5–7%) to reduce the risk of mediastinitis.	*Evidence level 2++. Strong recommendation, moderate quality of evidence*
	We recommend that patients should be encouraged to stop smoking at least 30 days prior to heart surgery.	*Evidence level 2+. Strong recommendation, moderate quality of evidence.*
	Obese or overweight patients should be encouraged to lose weight before surgery; we also recommend adjusting prophylactic antimicrobials doses, reinforce the preparation of the surgical field and ensure a very stable wound closure to avoid dehiscence, besides systematic closure with a NPWT device.	*Evidence level 2++. Strong recommendation, moderate quality of evidence.*
	When hair removal is considered necessary, we recommend the use of a depilatory cream or an electric razor, never a blade.	*Evidence level 1 +. Strong recommendation, high quality of evidence*
	We recommend knowing the state of *S. aureus* nasal carriage and proceeding with its eradication if possible or time allowable in positive patients before cardiac surgery.	*Evidence level 1−. Strong recommendation, moderate quality of evidence*
	We recommend PCR-based screening techniques for *S. aureus* carriage when a rapid method is necessary due to their high negative predictive value.	*Evidence level 1++. Strong recommendation, moderate quality of evidence*
	We recommend topical mupirocin for nasal decontamination in combination with chlorhexidine for skin decontamination.	*Evidence level 1+. Strong recommendation, high quality of evidence.*
	We suggest systematic decontamination in patients in whom nasal carrier status cannot be assessed in a timely manner.	*Evidence level 3. Strong recommendation, low quality of evidence*
	We recommend chlorhexidine over povidone-based products for skin preparation in cardiac surgery.	*Evidence level 2+. Strong recommendation, moderate quality of evidence*
	We recommend the control of blood glucose level during surgery (preferably with continuous insulin infusion) keeping it within 110 and 180 mg/dL.	*Evidence level 2+. Strong recommendation, moderate quality of evidence*
Diagnosis	Systematic collection of surveillance cultures at the time of closure of the mediastinal wound is not recommended	*Evidence level 2+. Strong recommendation, moderate quality of evidence*
	We recommend CT-guided puncture in patients with retrosternal collections, when there are no other means to confirm the aetiological diagnosis	*Evidence level 3. Strong recommendation, low quality of evidence*
	Cultures from sites that do not represent normally sterile tissues or fluids should be interpreted with caution, since they do not always allow determination of the causative agent of mediastinitis.	*Evidence level 3. Strong recommendation, low quality of evidence*
	Systematic epicardial pacing wire cultures are not recommended for early diagnosis of mediastinitis in the absence of clinical signs of infection.	*Evidence level 2+. Strong recommendation, moderate quality of evidence*
	Interpretation of bacterial culture results different from the original mediastinal tissue samples or blood cultures, and must be performed on a case-by-case basis. Their potential significance will depend on the type of isolated microorganism, the collection site and the clinical picture.	*Evidence level 3. Strong recommendation, low quality of evidence*
	Presence of significant bacteraemia, with no other clear origin in the 90 days after surgery, is potentially indicative of mediastinitis, particularly when the isolate is *S. aureus*.	*Evidence level 2+. Strong recommendation, low quality of evidence*
	There is not enough evidence yet to recommend the routine use of non-culture-based methods for the diagnosis of mediastinitis.	*Evidence level 3. Strong recommendation, low quality of evidence*
	Recommendations for the diagnostic approach in mediastinitis with classic negative culture cases should include: specific serological tests (*Coxiella* and *Bartonella*), deep mediastinal samples for 16S and 18S (panbacterial and panfungal, respectively) PCRs and cultures in special media for *Mycoplasma* spp., *Ureaplasma* spp., *Legionella* spp., *Nocardia* spp., Fungi and Mycobacteria.	*Evidence level 3. Strong recommendation, low quality of evidence*
	Plain X-rays are of limited use for the diagnosis of mediastinitis. We do not recommend their use as the first-choice diagnostic imaging test.	*Evidence level 2+. Strong recommendation, moderate quality of evidence*
	We recommend performing a CT scan as the first-choice diagnostic imaging technique in post-surgical mediastinitis. Scans should be performed two weeks after the surgery, when gas or normal collections of the immediate post-surgery period should not be present.	*Evidence level 2+. Strong recommendation, moderate evidence quality*
	We do not recommend a routine use of MRI, as there are few available data and wires can cause artefacts.	*Evidence level 3. Strong recommendation, low quality of evidence*
	Nuclear medicine techniques may be a useful tool in the study of sternum osteomyelitis. There is not enough evidence to recommend the routine use of PET-CT in patients with suspected mediastinitis. However, it may be useful in cases with suspected chronic infection, as well as for monitoring response to treatment.	*Evidence level 2+. Strong recommendation, moderate quality of evidence*
	We recommend performing a CT scan whenever there are signs of infection, despite the scarce information in the literature on this topic.	*Evidence level 3. Strong recommendation, low quality of evidence*
Surgical Management	The available evidence is not sufficient to recommend the use of topical antibiotics on the surgical site prior to closure.	*Evidence level 2+. Strong recommendation, with moderate quality of evidence*
	The use of surgical steel wires to close the sternum remains as the technique of choice. Superiority in reducing the incidence of mediastinitis has not been shown for other evaluated alternatives.	*Evidence level 1+. Strong recommendation, high quality of evidence*
	In high-risk patients, we recommend the use of prophylactic negative pressure wound therapy to reduce the incidence of infection.	*Evidence level 2+. Strong recommendation, moderate quality of evidence*
	We recommend the use of postoperative sternal immobilisation systems in all patients who undergo major cardiac surgery.	*Evidence Level 2+. Strong recommendation, moderate quality of evidence*
	Mediastinal lavage is not recommended on a routine basis, except in patients in whom Negative Pressure Wound Treatment (NPWT) cannot be performed or who require immediate closure. Povidone iodine should not be used in any case.	*Evidence level 2+. Strong recommendation, low quality of evidence*
	The surgical technique of choice in patients with post-surgical mediastinitis depends on the stage, sternal stability and bone viability. For wounds with minimal bone loss, relatively stable sternum, current evidence supports the use of NPWT as a first-line treatment or bridge for surgical closure or direct closure with muscle flap reconstruction if NPWT is not available. For unstable sternum and viable bone wound, we suggest that the initial application of NPWT followed by sternal rewiring or plates and coverage by muscle flaps. For wounds with unstable sternum and non-viable bone, Omental flaps coverage is recommended	*Evidence level 2+. Strong recommendation, low quality of evidence*
	To date, there is not enough evidence to recommend delaying surgical closure based on the persistence of positive wound cultures.	*Evidence level 3. Strong recommendation, low quality of evidence.*
	We recommend applying NPWT considering the following: the stability of the sternum, as a curative method (with or without surgery) in patients with a stable sternum, or as a bridge technique in preparation for surgery in subjects with an unstable sternum. NPWT should be checked every two to three days and last no longer than three weeks	*Evidence level 2+. Strong recommendation, moderate quality of evidence*
Medical Management	In adults with signs and symptoms of severe acute infection, we recommend initiating empirical antibiotic treatment as soon as there is clinical suspicion of mediastinitis. In non-critical adults, empirical treatment can wait for targeted treatment, based on laboratory findings.	*Evidence level 3. Strong recommendation, low quality of evidence.*
	In critically ill adult patients for whom aetiological confirmation is a threat, we recommend including methicillin-resistant *Staphylococcus* coverage for the empirical treatment of mediastinitis depending on local susceptibility pattern.	*Evidence level 4. Conditioned recommendation based on expert opinion. Very low level of evidence*
	In the empirical treatment of adult patients with PSM, we recommend including coverage against Gram-negative bacilli considering local epidemiology, until aetiological confirmation is available.	*Evidence level 2−. Strong recommendation. Low level of evidence*
	We do not recommend systematic administration of antifungal treatments as part of the empirical treatment of PSM. It should only be administered in critical situations where there are risk factors for invasive fungal infection.	*Evidence level 4. Conditioned recommendation based on expert opinion. Very low level of evidence*
	With the current available information, it is not possible to recommend mediastinal irrigation either with antibiotics or antiseptic substances.	*Evidence level 3. Strong recommendation, low quality of evidence*
	We recommend the use of sequential antimicrobial treatment in stable patients who have received adequate surgical treatment after a period of i.v. therapy, the proper duration of which is difficult to determine. Active antimicrobials with high bioavailability have been used, depending on the aetiology.	*Evidence level 2−. Strong recommendation. Low level of evidence*
	In patients with methicillin-sensitive staphylococcal mediastinitis, beta-lactam drugs such as isoxazolyl penicillins or cefazolin are preferred	*Evidence level 3. Strong recommendation, low quality of evidence*
	In patients with methicillin-resistant staphylococcal infections, the use of glycopeptides or glycolipopeptides (vancomycin or daptomycin) is recommended.	*Evidence level 3. Strong recommendation, low quality of evidence*
	In Enterococcal mediastinitis, it is recommended to follow the accepted scheme for endocarditis with double beta-lactam treatment (ampicillin + ceftriaxone) or the beta-lactam-aminoglycoside combination.	*Evidence level 3. Strong recommendation, low quality of evidence*
	The selection of antimicrobial treatment in patients with proven GNB mediastinitis must be adjusted in each circumstance and under expert supervision.	*Evidence level 3. Strong recommendation, low quality of evidence*
	Confirmed mediastinitis caused by fungi is a very serious rare entity. The treatment is only indicative (described in Table 4) and always requires expert consensus.	*Evidence level 3. Strong recommendation, low quality of evidence*
	We recommend an average duration of four to six weeks in bacterial mediastinitis. When possible, we recommend switching to oral antimicrobials at week two to three if feasible. In case of sternal osteomyelitis and/or fungal mediastinitis, we recommend prolonged treatment.	*Evidence level 4. Conditioned recommendation based on expert opinion. Very low-grade quality of evidence*
